# Investigation of actual exposure to facial sheet mask preceding its risk assessment

**DOI:** 10.1038/s41598-022-05351-3

**Published:** 2022-01-24

**Authors:** Lihong Zhou, Jian Chen, Tian Chen, Huailong Chang, Wenguang Cui, Yuanyuan She, Zhu Li, Wenhua Tang, Dengfeng Yuan, Zhitao Chen, Jin Su

**Affiliations:** 1 NMPA Key Laboratory for Monitoring and Evaluation of Cosmetics, Shanghai Municipal Center for Disease Control and Prevention, 1380 Zhongshan Rd. W., Changning District, Shanghai, 200336 China; 2 Shanghai Jahwa United Co. Ltd., Shanghai, 200082 China; 3 Division of Public Health Service and Safety Assessment, Shanghai Institute of Preventive Medicine, 1380 Zhongshan Rd. W., Changning District, Shanghai, 200336 China

**Keywords:** Risk factors, Preventive medicine

## Abstract

The present study aimed to reveal the amount per application of facial sheet masks and its influencing factors in Chinese population to form the base for an accurate exposure assessment. A total of 175 healthy subjects aged 18 years or older were recruited and divided into two subgroups: one group of 35 subjects were asked to apply same mask for 5, 10, 15, 20, 25, and 30 min respectively, and the other 140 subjects were instructed to apply one of four types of facial sheet masks presented in the market for 15 min. Furthermore, phenoxyethanol and methylparaben were measured to reflect actual exposure to chemicals. The sharp increase in the relative exposure to phenoxyethanol (CAS NO.122-99-6) and methylparaben at 25 min and longer suggests applying facial sheet masks for longer than 20 min may drive the exposure to hazardous chemicals to increase significantly. The 90th percentile of amount per application for plant-cellulose, bamboo charcoal fiber, bio-cellulose, and binchotan charcoal fiber-based masks was 5.753, 5.371, 5.017, and 4.821 g respectively. In addition, men and subjects with sebaceous skin demonstrated lower amount per application compared to women and subjects with dry skin, respectively. Finally, our data showed that the larger the contacting area between face and mask, the more amount per application. We concluded that the appropriate time of application should be less than 20 min. And mask fabrics, gender, sebum content, and contacting area could significantly impact the risk assessment of facial sheet masks. Our data for the first time provides insights into a realistic risk assessment of facial sheet masks in Chinese population.

## Introduction

The cosmetic industry is currently experiencing a record-breaking high-speed growth in China. According to the National Bureau of Statistics of China, the total retail sale of cosmetics reached 299.2 billion Chinese Yuan in 2019, an increase of 12.6% over the previous year^1^. In concordance with this rapid increase in the market size is the rise of safety concerns in consumers and the need for regulatory entities to accurately assess and appropriately manage the risks concerning the use of cosmetic products. In response to this emerging need, the China National Medical Products Administration has recently issued a new Technical Guideline- of Cosmetic Safety Assessment, which provides detailed instructions on the safety assessments of cosmetic ingredients and finished products. However, little is known about actual exposure to cosmetics because of the lack of data on consumption by Chinese consumers.

In order to perform a realistic risk assessment of cosmetic products, it is crucial to acquire the consumption data in terms of frequency of use, amount used per application, dermally contacted area, etc. Several studies have been conducted in Europe focusing on the amount of consumption of various types of cosmetic products, the results of which have been adopted by the Scientific Committee on Consumer Safety (SCCS) in its Notes of Guidance on safety assessment of cosmetics to guide risk assessment of raw ingredients in Europe^2–5^. In the United States, even though the Food and Drug Administration (USFDA) does not require the cosmetic industry to provide safety assessment data for the purpose of product registration, studies have still been carried out to reveal consumer’s consumption of cosmetic products, including large scale investigations and epidemiological studies^6–9^. However, those types of studies focusing on the Chinese population remain limited. There is only one study reporting the amount of exposure to phthalates from use of personal care products in Chinese population^10^. Therefore, there is basically no available human consumption data specifically refined to the Chinese population can be used for risk assessment of cosmetics in one of the world’s largest markets.

Facial masks are increasingly used in China by both men and women. The Chinese facial mask market experienced an annual growth rate of 20.1% and reached 31.5 billion Chinese Yuan in 2019^11^. Furthermore, it is projected that with a compound annual growth rate of 15.4% in the next 5 years, the total market value shall reach 64.5 billion Chinese Yuan by 2024^11^. However, even for a market of such large scale, lack of data on the use of this specific type of cosmetic product by the Chinese consumers still exists, though many investigations have been conducted in the developed countries^12–14^. Given that the compositions of mask used by and skin conditions and lifestyles of Chinese people varied dramatically from those in the western world, it is logic to infer that the consumption of facial masks in China is significantly different. Moreover, incidences of skin problems including irritancy and allergic contact dermatitis have been reported in China due to misuse of facial sheet masks such as longer than anticipated time. Thus, it is urgent and of vital importance to obtain data on the consumption of facial masks in the Chinese population in order to perform an accurate risk assessment of this type of product in this specific country.

The aim of the current study is to enhance and complement the existing data in Europe and the US with data from China, and to provide basic information for future risk assessment of sheet masks in the Chinese population. Therefore, data on amount per application of facial sheet were obtained using 4 different kinds of fabrics presented in the Chinese cosmetics market by controlled human application tests with different application-time scenarios in addition to a clinical condition survey. To our best knowledge, this is the first study to investigate the potential amount of exposure to facial sheet masks and the corresponding impacting factors in the Chinese population.

## Results

### General characteristics of the subjects

The general characteristics of the test subjects in our study were summarized in Table 1. The amount per application data obtained from one subject was identified as an outlier and was excluded from analysis, leaving the total number of subjects qualified for analysis to 139. Of these 139 subjects, 30 (21.6%) were men and 109 (78.4%) were women. The overall mean age of the subjects was 44.0 ± 9.7 years old. The skin types reported by subjects consisted of 37 Normal (26.6%), 67 Combination (48.2%), 21 Dry (15.1%), and 14 Oily (10.1%). The pH of the facial skin of the subjects averaged at 5.16 ± 0.39 and all fell into the normal range. We also tested the sebum and water content of the facial skin of the subjects. The overall mean sebum content of the subjects was 34.3 ± 26.1 au (arbitrary unit), and the water content of the forehead and cheek was 50.7 ± 10.5 au and 39.4 ± 10.0 au, respectively. There was no clear indication of a difference in the distribution of the subjects between mask types.

**Table 1:** General characteristics of the subjects [*n* (%) or mean ± SD].

Characteristics	Overall (*N* = 139)	BC (*n* = 35)	PC (*n* = 35)	BCF (*n* = 34)	BioC (*n* = 35)
**Gender**					
Male	30 (21.6)	8 (22.9)	8 (22.9)	7 (20.6)	7 (20.0)
Female	109 (78.4)	27 (77.1)	27 (77.1)	27 (79.4)	28 (80.0)
Age	44.0 ± 9.7	43.7 ± 9.3	45.8 ± 9.9	42.4 ± 10.8	44.2 ± 8.9
**Self-reported skin type**					
Normal	37 (26.6)	9 (25.7)	12 (34.3)	7 (20.6)	9 (25.7)
Combination	67 (48.2)	15 (42.9)	14 (40.0)	15 (44.1)	23 (65.7)
Dry	21 (15.1)	8 (22.9)	6 (17.1)	5 (14.7)	2 (5.7)
Oily	14 (10.1)	3 (8.6)	3 (8.6)	7 (20.6)	1 (2.9)
pH	5.16 ± 0.39	5.19 ± 0.36	4.99 ± 0.41	5.21 ± 0.30	5.27 ± 0.42
Forehead sebum (au)	34.3 ± 26.1	35.3 ± 24.2	31.3 ± 22.0	42.0 ± 26.9	28.8 ± 29.8
Forehead water content (au)	50.7 ± 10.5	45.9 ± 10.2	51.3 ± 12.5	53.4 ± 8.1	52.4 ± 9.7
Cheek water content (au)	39.4 ± 10.0	38.3 ± 11.3	40.8 ± 9.4	37.2 ± 9.1	41.2 ± 9.8
**Folds occurrence**					
Yes	68 (48.9)	9 (25.7)	18 (51.4)	20 (58.8)	21 (60.0)
No	71 (51.1)	26 (74.3)	17 (48.6)	14 (41.2)	14 (40.0)
**Area of mask**					
≤ Area of face	91 (65.5)	27 (77.1)	25 (71.4)	17 (50.0)	22 (62.9)
> Area of face	48 (34.5)	8 (22.9)	10 (28.6)	17 (50.0)	13 (37.1)
Amount per application (g)	4.780 ± 0.506	4.452 ± 0.361	5.348 ± 0.367	4.765 ± 0.423	4.553 ± 0.320
P90 (g)	5.425	4.821	5.753	5.371	5.017

*BC* binchotan charcoal fiber, *PC* plant cellulose, *BCF* bamboo charcoal fiber, BioC bio-cellulose.

### Time trend of amount per application

**Figure 1:**
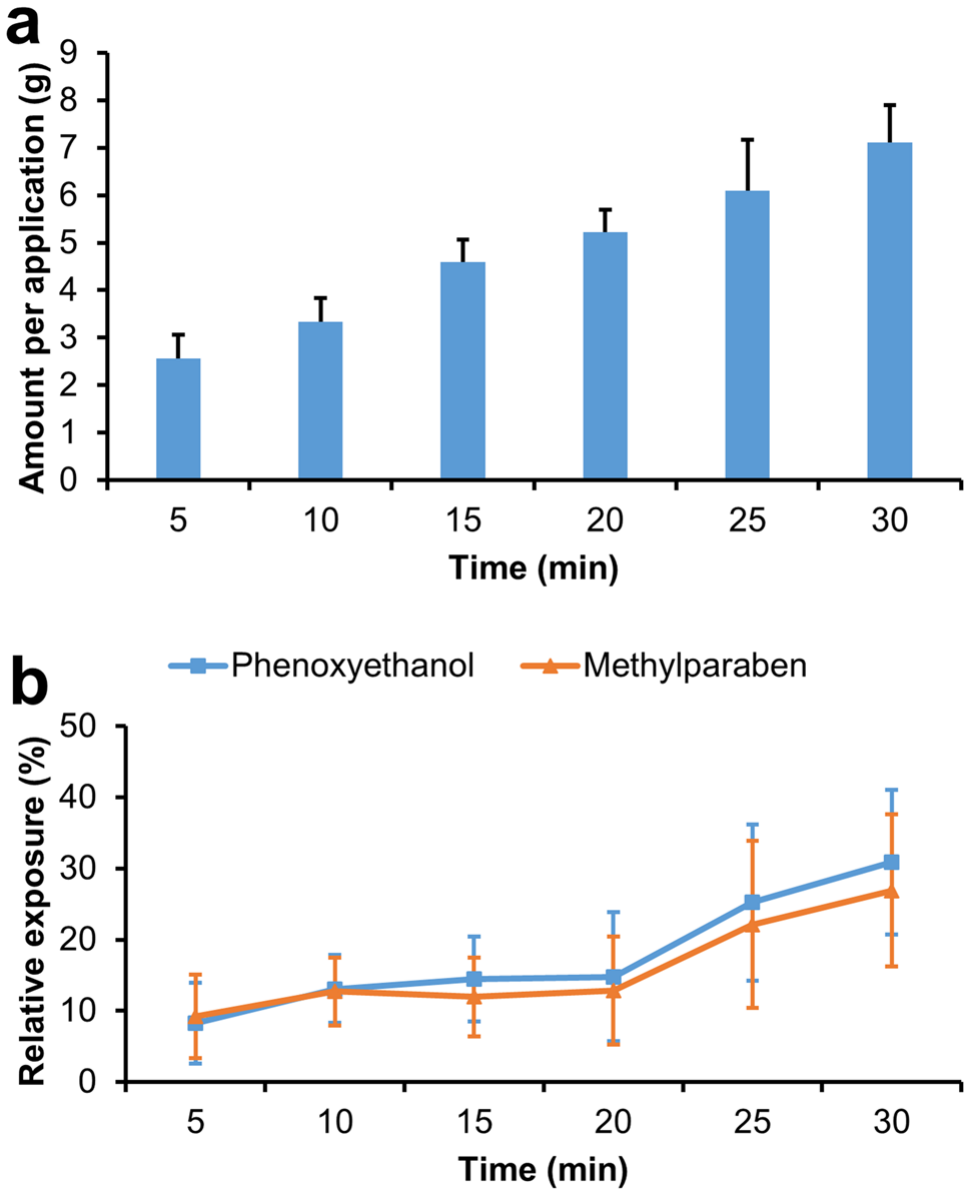
The time trends of amount per application of facial sheet mask and the relative exposure amount to non-VOCs represented by phenoxyethanol and methylparaben. A subpopulation of the subjects was asked to apply the masks for 5, 10, 15, 20, 25, and 30 min respectively and the residues were collected for HPLC analysis and were compared to the amount measured in the masks of same batch and size before use. The amount per application at different time of duration was shown in (**a**); and relative exposure to phenoxyethanol and methylparaben was calculated and expressed as percentage of the difference between the content after use and before use over their initial content before use and was shown in (**b**). Data was shown as mean ± SD.

We first tested the trend of amount per application over different application time in the plant cellulose based facial sheet mask, which was randomly chosen from the four types of mask. As shown in Fig. 1a, the amount per application gradually went up from 2.567 ± 0.496 g to 7.108 ± 0.796 g following prolonging the application time from 5 to 30 min. Upon understanding the time pattern of amount per application of facial sheet masks, we intended to further know whether this pattern truly reflected the amount of exposure to chemicals in the masks. Since phenoxyethanol and methylparaben are among the most commonly used preservatives in personal care products that can induce contact dermatitis in consumers^15,16^, we chose both chemicals as indicators of exposure to hazardous chemicals. Relative exposure to each preservative was calculated and expressed as percentage of the difference in the content between after and before use over their initial content before use. As shown in Fig. 1b, consistent with the increase in application time, the relative exposure to both preservatives gradually went up. Moreover, the relative exposure to both preservatives increased sharply when the application time reached 25 min or longer (25.2% and 22.1% at 25 min for phenoxyethanol and methylparaben respectively), compared to that of 20 min or shorter (14.8% and 12.8% at 20 min for phenoxyethanol and methylparaben respectively) (Fig. 1b). Thus, from a safety perspective, applying masks for more than 20 min should not be recommended.

### Factors affecting the amount per application

Out of the four types of masks, one recommended an application time of 15 min, one of 10–15 min, and two of 15–20 min on the labels of the masks. Therefore, 15 min was designated to be the application time in our comparison study between the four masks. During the test, in case of 68 out of 139 subjects (48.9%), the masks applied to the subjects presented folds on the mask. The area of the masks was larger than that of the face of the corresponding subjects in the case of 48 out of 139 subjects (34.5%) (Table 1). The overall mean and 90th percentile of amount per application was 4.780 ± 0.506 g and 5.425 g respectively (Table 1). After the impacts of ambient factors were controlled by putting all the subjects in a room with constant temperature and humidity, we tested the influences of potential factors in addition to time on the amount per application. This was realized by fitting mask fabric, gender, age, self-reported skin type, pH, sebum content, forehead and cheek water content, folds occurrence during use, and fitness of the mask into our model. As displayed in Table 2, our model showed statistical significance (*F* = 21.717, *P* < 0.001), indicating that at least part of the factors included in the model could impact the amount per application. Further analysis of the coefficients showed that mask fabrics, gender, facial sebum, fold occurrence, and fitness of the mask may significantly impact the exposure per application.

**Table 2:** Results of the hypothesis test of the linear regression model.

	Sum of squares	df	Mean square	F	Sig.
Regression	25.063	14	1.79	21.717	.000
Residual	10.222	124	0.082		
Total	35.285	138			

### Mask fabrics

The mean amount per application for binchotan charcoal fiber (BC), plant cellulose (PC), bamboo charcoal fiber (BCF), and bio-cellulose (BioC) based masks were 4.452 ± 0.361, 5.348 ± 0.367, 4.765 ± 0.423, and 4.553 ± 0.320 g respectively (Table 1). Table 3 showed that the coefficients for BC, PC, and BCF based masks were − 0.212 [− 0.363, − 0.061] (*P* = 0.006), 0.748 [0.604, 0.892] (*P* < 0.001), and 0.214 [0.068, 0.361] (*P* = 0.005) respectively, suggesting that the influences of the mask fabrics on the amount per application were significant. In our model, BioC based mask was randomly selected to be the reference mask. The coefficient for BC based mask means that compared to the reference BioC based mask, BC based mask was estimated to induce a mean reduction of 0.212 g of amount per application when other factors were kept at constant levels. Similarly, application of PC and BCF based masks were estimated to increase the mean amount by 0.748 g and 0.214 g respectively. Together, the order of amount per application over different mask fabrics in our study was BC < BioC < BCF < PC.

**Table 3:** Coefficients of the variables in the linear regression model and their corresponding hypothesis test results.

	Unstandardized coefficients	Std. error	Standardized coefficients	t	Sig.	95.0% Confidence interval for B	
	B		Beta			Lower bound	Upper bound
(Constant)	4.888	0.417		11.731	0.000	4.063	5.712
Gender*	− 0.416	0.069	− 0.34	− 6.002	0.000	− 0.554	− 0.279
Age	0.002	0.003	0.041	0.734	0.464	− 0.004	0.008
pH	0.036	0.069	0.028	0.523	0.602	− 0.101	0.173
Sebum*	0.003	0.001	0.155	2.778	0.006	0.001	0.005
F. water	− 0.003	0.003	− 0.058	− 1.018	0.310	− 0.008	0.003
C. water	0.002	0.003	0.038	0.696	0.488	− 0.004	0.008
Fold*	− 0.125	0.063	− 0.124	− 1.988	0.049	− 0.249	− 0.001
Area*	0.149	0.064	0.14	2.324	0.022	0.022	0.275
Skin type1	0.138	0.102	0.121	1.359	0.177	− 0.063	0.339
Skin type2	0.069	0.097	0.069	0.718	0.474	− 0.122	0.261
Skin type3	0.134	0.111	0.096	1.212	0.228	− 0.085	0.354
BC*	− 0.212	0.076	− 0.183	− 2.786	0.006	− 0.363	− 0.061
PC*	0.748	0.073	0.644	10.3	0.000	0.604	0.892
BCF*	0.214	0.074	0.183	2.889	0.005	0.068	0.361

*BC* binchotan charcoal fiber, *PC* plant cellulose, *BCF* bamboo charcoal fiber.

*P < 0.05.

### Gender

The coefficient for gender in our model was − 0.416 [− 0.554, − 0.279] (*P* < 0.001), suggesting that the amount per application varied significantly between men and women. The coefficient of − 0.416 in this case means that when other factors were kept at constant levels, application of masks to men were estimated to decrease the mean amount by 0.416 g compared to that in women.

### Facial sebum

The coefficient for facial sebum content in our model was − 0.003 [0.001, 0.005] (*P* = 0.006), indicating that facial sebum content may also have significant effects on the amount per application. The number − 0.003 means that in case levels of other factors were set up, every single unit of increase in the facial sebum content can decrease the estimated mean amount per application by 0.003 g.

### Contacting area

The coefficients for fold occurrence and fitness of the mask were − 0.125 [− 0.249, − 0.001] (*P* = 0.049) and 0.149 [0.022, 0.275] (*P* = 0.022) respectively, which indicated that if there was/were fold(s) presented on the mask during use, the estimated mean amount per application can be decreased by 0.125 g and if the mask could be fully stretched and applied to the face, the estimated mean amount can be increased by 0.149 g compared to that of partially stretched and applied masks. Collectively, those two factors reflected that the amount per application went up when the contacting area between the mask and face increased.

## Discussion

In the present study, we for the first time investigated the amount per application of facial sheet masks when applied for different lengths of time and the potential factors that might influence the consumption amount by the Chinese consumers. Our results demonstrated that the appropriate time of application of facial sheet masks may be less than 20 min, at which point we believe the exposure to chemicals were kept at low level. We also showed that mask fabrics, gender, facial sebum content, and contacting area between face and the mask might impact the amount per application. According to the SCCS Notes of Guidance for the Testing of Cosmetic Ingredients and Their Safety Evaluation (11th revision)^5^, the calculation of systemic exposure dose (SED) requires amount per application, frequency of application, concentration, and dermal absorption percentage. Since the concentration of chemicals is known at formulation design stage and the dermal absorption can be defaulted at 50% (or 100% for safety consideration), what matters the most now is only the amount per application, which is exactly the focus of our study. Thus, our data provides a solid base for future assessment of risks imposed to the Chinese consumers by use of sheet masks.

The overall mean amount per application in the current study was 4.780 ± 0.506 g, which was slightly lower (3.4%) than that in the Japanese population reported by Yamaguchi et al. in 2017 (4.95 ± 2.21 g)^14^. This consistency in the amount per application between two different studies indicates that users of same race, east Asian in this case, with similar skin conditions and lifestyles may have comparable exposure amounts from facial sheet masks. Thus, our findings may be generalized for guiding the risk assessment of facial sheet masks across Asia–pacific countries and regions like China, Japan, and Korea. However, the 90th percentile of amount per application, which is commonly adopted in a practical risk assessment, was 5.425 g in the present study, lower than 7.19 g reported in Yamaguchi study^14^. It is noteworthy that the variation of the data in the Japanese study (SD = 2.21) was much larger than that of the current Chinese study (SD = 0.506), despite the Japanese study had more subjects (*N* = 263) than the current study (*N* = 139). Comparing the designs of two studies, it is noticeable that the types of masks applied were not controlled in the Japanese study, which at least in part explained the large variation in data. While the Japanese study reflected the real-life scenarios as claimed by the authors, this might mitigate the influences of mask types on the consumption amount.

Facial sheet fabrics can vary significantly in their mechanical properties and abilities to retain the ingredients and/or to release them into consumer skin^17^. However, no data has been published regarding the potential impacts of mask fabrics on the amount per application from a safety perspective. Our results showed that when consumers switched from BC-based masks to PC-based masks, the mean exposure amount per application increased by more than 20% (4.452 g versus 5.348 g). Thus, we suggest that safety assessors conducting risk assessment of their products should take the materials of the fabrics into consideration before putting them into the market. In addition, from a safety point of view, we also recommend to use the 90th percentile for PC based masks (5.753 g) to be adopted as a highly conservative exposure value in a realistic exposure as well as risk assessment.

The physiological conditions of men’s skin are different from those of women in many aspects, including hormone metabolism, hair growth, sweat rate, sebum production, surface pH, and fat accumulation^18^. Therefore, it is imaginable that men and women may differ in amount of exposure even when apply the masks in the same exposure scenarios. Indeed, our data suggested that the amount per application differed significantly between men and women. In our study, men’s exposure to facial sheet masks was suggested to be lower than women’s if other factors were controlled. In addition, our study demonstrated that the facial sebum content may significantly impact the exposure to facial sheet masks, which might contribute to part of the gender difference in the exposure amount, i.e., there might be collinearity between facial sebum content and gender. However, the variance inflation factor (VIF), a measure of collinearity, in our model was < 10 (data not shown), suggesting that the probability of multicollinearity was low. Thus, the effects of sebum content on amount per application did exist in addition to gender differences.

The sheets are commonly one-size-fits-all, whereas every face is not of the same size or shape. Thus, consumers with various size of face shall have different contacting area with the mask. Plus, the occurrence of folds or bubbles on the sheet during use is practically unavoidable, which may further contribute to the difference in contacting area. The contacting area of the skin is of key importance in terms of determining the exposure amount in risk assessment of dermally exposed substances in addition to frequency of use and amount used per application^5^. In consistence with this common sense, both the fitness of masks to the face and the occurrence of folds during use significantly influenced the exposure amount in our study. Although it might be too complex to account for face size and fold occurrence in a realistic risk assessment, those factors might help to derive conservative estimates.

In addition to amount per application and frequency of application, application time is another key factor that decides the exposure amount in risk assessment. While most facial sheet masks recommend a 15–20 min application time, the scientific rationale behind this recommendation is still unknown. It is easy to accept that an appropriate application time means at which point the absorption of active ingredients reaches a peak while the exposure to hazardous substances remains at low level. From this perspective, our data may for the first time provides a scientific base for recommending the appropriate application time. In the present study, applying facial sheet masks for longer than 20 min drove the exposure to preservatives to a dramatic increase. This further reminded us that in a realistic risk assessment, use of amount per application may underestimate the exposure to facial sheet masks if masks are applied for longer than 20 min. Therefore, we not only recommend consumers to apply facial sheet masks for less than 20 min to ensure safety, but also risk assessors to take caution in assessing risks associated with an application time of longer than 20 min. We hypothesized that the evaporation of water when applying the masks on the face made preservatives concentrate in the mask, and when the application time went up to more than 25 min, the skin started to absorb chemicals fast from the sheet due to the concentration difference between the sheet and facial skin.

We concluded that Chinese consumers’ exposure to facial sheet masks depended on the mask fabrics, gender, facial sebum content, and the contacting area with the mask. The 90th percentile of amount per application for PC based masks (5.753 g), which was the worst case in the present study, may be used in a realistic risk assessment of facial sheet masks for Chinese consumers in order to perform a conservative risk assessment. Applying for less than 20 min is recommended to be appropriate for facial sheet masks. The current study provides firm basis for exposure assessment of facial sheet masks, and extra exposure information regarding gender and skin sebum content could be of importance when a specific population requires attention.

## Methods

### Survey

In order to acquire data on the exposure frequency from consumers with different genders, ages, and skin conditions, subjects randomly selected from network communities were asked to complete a survey on their application patterns. The contents of the survey consisted of information on demographics, types of daily used masks, and application patterns. In addition, subjects recruited to participate the tests were also asked to complete a paper form of the survey.

### Masks

Binchotan charcoal fiber (BC), plant cellulose (PC), bamboo charcoal fiber (BCF), and bio-cellulose (BioC) based facial sheet masks, which have already been registered and put into the market in China, were provided by Shanghai Jahwa United Co., Ltd. (Shanghai, China). For each kind of masks, products of the same brand, size, and batch number were selected. The selected masks were then subjected to evaluation in compliance with relative regulations, of which qualified masks were selected as test materials in the current study. The facial cleanser applied in the test was provided by the same supplier and was also subjected to evaluation in compliance with relative regulations before those qualified were selected.

### Subjects

The present study was reviewed and approved by Shanghai Ethics Committee for Clinical Research. All research was performed in accordance with relevant guidelines and regulations. A total of 175 subjects aged at older than 18 years were recruited with no special requirement for gender. Informed consent was obtained from all human subjects. Subjects were equally assigned into each group based on their self-reported skin conditions. Subjects with following conditions were excluded from the test:

1. Users of anti-histidine medicines in the immediate week or users of immune repressive agents in the immediate month before test.
2. Users of anti-inflammatory medicines on the test site in the past 2 months.
3. Patients with unhealed inflammatory skin diseases.
4. Insulin dependent diabetic patients.
5. Patients with asthma or other chronic respiratory conditions undergoing treatments.
6. Users of anti-tumor chemo-therapeutic agents in the past 6 months.
7. Patients with immuno-deficiency or auto immune diseases.
8. Breeding or pregnant women.
9. Subjects with both sides of breast and lymph nodes incised.
10. People with scars, pigments, shrinkage, bright erythema or other deficiencies that may interfere the judgement of test results on the test site.
11. Subjects participating in other clinical trials.
12. Highly sensitive people.
13. Not voluntarily participated or may not be able to complete the test procedure in compliance with the test requirements.

### Test procedure

For testing the amount per application in different application-time scenarios, a group of 35 subjects were recruited. Each subject was requested to complete 6 tests in total with the only variation in the application time, i.e., 5, 10, 15, 20, 25, and 30 min, in 6 not necessarily consecutive days. All the tests were accomplished in 2 months. For testing the potential factors that impact the amount per application, 140 subjects were recruited and randomly assigned to apply one of the four different kinds of facial sheet masks.

All the tests were carried out in a room with constant ambient temperature (20–25 ℃) and humidity (40–55%). On the day of testing, subjects were first allowed to accommodate to the ambient conditions for at least 30 min before started, during which subjects were asked to complete a questionnaire on their application patterns. Subjects were then given facial cleanser to clean their faces, after which subjects were tested for facial sebum and water content with a Cutometer Dual MPA580 (Courage + Khazaka electronic GmbH, Köln, Germany). For each test, subjects were given coded masks randomly and applied the masks in accordance with instructions. Detailed information including whether the mask was large enough to cover the whole face and whether fold(s) occurred during the test procedure was recorded. Facial sheet mask together with the corresponding package was weighed before and after application. For subjects reported to also apply the essence left in the package after mask application (e.g., on the neck or back of hand), they were requested to use the essence as usually do before weighing the masks and the package.

### High-performance liquid chromatography

After testing for amount per application over different application time, the fabrics and any residual liquid left in the package were collected, into which methanol was added until the final volume reached 100 mL. The chromatographic analysis of phenoxyethanol and methylparaben was performed using an RP-HPLC system (WATERS, Milford, MA) equipped with an autosampler and a photo-diode array (PDA) detector. The column configuration consisted of a ZORBAX SB-C18 (250 mm × 4.6 mm i.d., 5 μm particle size) (Agilent Technologies, Santa Clara, CA). UV absorption was measured at 254 nm. Socratic separation was carried out with three mobile phases composed of methanol, acetonitrile, and water (30:10:60). The flow rate was kept at 1.0 mL/min and the sample injection volume was 10 μL. Data was analyzed by the Empower Chromatography Data System (version 3.0). Relative exposure was calculated and expressed as percentage of the difference between the content of each preservative after use and before use over their initial content before use.

### Statistical analyses

The amount per application of a single facial sheet mask was calculated by subtracting the weight of the mask and package after application from the weight before application. Data was expressed as mean ± standard deviation (SD). Factors that may have impacts on the amount per application were fitted into a multi-factorial linear regression model, in which dummy variables were coded for self-reported skin type and mask fabric with oily skin and bio-cellulose based mask set as the reference group respectively. All analyses were carried out using SPSS version 26.0 (IBM SPSS Statistics, Armonk, NY). *P* ≤ 0.05 was taken as statistically significant.

### Ethics declaration

The present study was reviewed and approved by Shanghai Ethics Committee for Clinical Research.

## Data Availability

Data is available upon request.
